# Analyzing Transverse Momentum Spectra of Pions, Kaons and Protons in *p*–*p*, *p*–A and A–A Collisions via the Blast-Wave Model with Fluctuations

**DOI:** 10.3390/e23070803

**Published:** 2021-06-24

**Authors:** Hai-Ling Lao, Fu-Hu Liu, Bo-Qiang Ma

**Affiliations:** 1 Center for High Energy Physics, Peking University, Beijing 100871, China; 2 State Key Laboratory of Quantum Optics and Quantum Optics Devices, Collaborative Innovation Center of Extreme Optics, Institute of Theoretical Physics, Shanxi University, Taiyuan 030006, China; 3 State Key Laboratory of Nuclear Physics and Technology, School of Physics, Peking University, Beijing 100871, China; 4 Collaborative Innovation Center of Quantum Matter, Beijing 100871, China

**Keywords:** high energy nucleus–nucleus collisions, transverse momentum spectra, kinetic freeze-out temperature, transverse flow velocity, proper time, the blast-wave model with fluctuations, 13.75.Cs, 13.85.Fb, 25.75.Cj

## Abstract

The transverse momentum spectra of different types of particles, $\pi^{\pm }$, $K^{\pm }$, *p* and $\bar{p}$, produced at mid-(pseudo)rapidity in different centrality lead–lead (Pb–Pb) collisions at 2.76 TeV; proton–lead (*p*–Pb) collisions at 5.02 TeV; xenon–xenon (Xe–Xe) collisions at 5.44 TeV; and proton–proton (*p*–*p*) collisions at 0.9, 2.76, 5.02, 7 and 13 TeV, were analyzed by the blast-wave model with fluctuations. With the experimental data measured by the ALICE and CMS Collaborations at the Large Hadron Collider (LHC), the kinetic freeze-out temperature, transverse flow velocity and proper time were extracted from fitting the transverse momentum spectra. In nucleus–nucleus (A–A) and proton–nucleus (*p*–A) collisions, the three parameters decrease with the decrease of event centrality from central to peripheral, indicating higher degrees of excitation, quicker expansion velocities and longer evolution times for central collisions. In *p*–*p* collisions, the kinetic freeze-out temperature is nearly invariant with the increase of energy, though the transverse flow velocity and proper time increase slightly, in the considered energy range.

## 1. Introduction

In high-energy collisions, one of the most important questions is the identification of various phases of dense matter. Quark-gluon plasma (QGP) [1,2,3], which is considered a new state of matter, was produced in the early universe shortly after the Big Bang, prior to the condensation in hadrons. High energy nucleus–nucleus (heavy ion) collisions at the large hadron collider (LHC) [4,5,6,7,8] provide another excellent environment with high temperatures and high density under which QGP are expected to form and to live for a longer lifetime than that at the relativistic heavy ion collider (RHIC) [9]. Presently, the LHC has performed four different types of collisions, proton–proton (*p*–*p*), proton–lead (*p*–Pb), lead–lead (Pb–Pb) and xenon–xenon (Xe–Xe) collisions, at different collision energies. The former two with low multiplicity are not expected to form QGP due to small systems and less energy deposition, though the deconfinement of quarks and gluons may appear. The latter two are expected to form QGP due to large systems and high energy. It is possible that the former two with high multiplicity will also form QGP due to violent collisions.

Kinetic freeze-out temperature, transverse flow velocity and proper time are three important parameters with which to characterize the thermal properties of different stages during high-energy *p*–*p*, proton–nucleus (*p*–A) and nucleus–nucleus (A–A) collisions. The proper time reflects the time elapsed from the initial collisions to the stage of kinetic freeze-out. A large proper time represents that the interacting system has a long life. Temperature is an important concept in the physics of high-energy collisions. Usually, papers [6,10,11,12,13] use four types of temperature: initial temperature, chemical freeze-out temperature, kinetic freeze-out temperature and effective temperature. Firstly, the initial temperature describes the excitation degree of the interacting system at the initial stage of collisions. Secondly, the chemical freeze-out temperature describes the excitation degree of the interacting system at the stage of chemical equilibrium, where the chemical components (relative fractions) of particles are no longer changed. Thirdly, the kinetic freeze-out temperature describes the excitation degree of the interacting system at the stage of kinetic and thermal equilibrium in which the (transverse) momentum spectra of particles are no longer changed. Fourthly, the effective temperature is not a real temperature; it is related to particle mass and describes the sum of the excitation degree of the interacting system and the effect of transverse flow at the stage of kinetic freeze-out, where the transverse flow resulted from the impact and squeeze reflects the hydrodynamic expansion of the interacting system.

Generally, since the initial stage of collisions happens earlier than (or alongside) the stage of chemical freeze-out, the initial temperature is larger than (or equal to) the chemical freeze-out temperature. The chemical freeze-out temperature is equal to or larger than the kinetic freeze-out temperature, as the chemical equilibrium comes about at the same time as or before the kinetic equilibrium. It is usually obtained from the particle ratios [14,15,16]. The effective temperature is larger than the kinetic freeze-out temperature due to mass and flow effects [17,18]. It can be extracted from the transverse momentum spectra by using some distribution laws, such as the standard (Boltzmann, Fermi–Dirac and Bose–Einstein) distributions, the Tsallis distribution and so forth. The kinetic freeze-out temperature can be extracted from the transverse momentum spectra using models such as the blast-wave model with Boltzmann–Gibbs statistics, the blast-wave model with Tsallis statistics and the blast-wave model with fluctuations. Usually, the initial, chemical freeze-out and kinetic freeze-out temperatures are real temperatures.

We were very interested in studying the transverse excitation, the dynamic expansion characteristic and the lifetime of the interacting system, as their relation is important for mapping the phase diagram, and with them we could obtain some information about the proper time. In order to extract some quantities and study their dependences on event centrality and collision energy, we may use some models to analyze the particle spectra. These models include, but are not limited to, the blast-wave model with Boltzmann–Gibbs statistics [19,20], the blast-wave model with Tsallis statistics [21,22,23] and the alternative method [24,25,26,27,28] based on the standard distribution (including Boltzmann, Fermi-Dirac and Bose–Einstein distributions) or Tsallis distribution. The blast-wave model is a traditional and current method; it has very widespread applicability. The model makes the simple assumption that particles are locally thermalized in a hard-sphere, uniform-density source at a kinetic freeze-out temperature and are moving with a common collective manner in a transverse radial flow velocity field. In the alternative method, the kinetic freeze-out temperature is regarded as the intercept in the linear relation between effective temperature and rest mass, and the transverse flow velocity is regarded as the slope in the linear relation between mean transverse momenta and mean energy. Like the alternative method, too, the traditional blast-wave model can only extract the kinetic freeze-out temperature and transverse flow velocity. By inheritance and through development, the blast-wave model with fluctuations can extract the kinetic freeze-out temperature, transverse flow velocity and proper time simultaneously.

To understand the thermal properties of different stages of high energy collisions, we applied the blast-wave model with fluctuations to study the transverse momentum ($p_{T}$) spectra of different particles produced in A–A, *p*–A and *p*–*p* collisions at the LHC. The kinetic freeze-out temperature ($T_{0}$), transverse flow velocity ($\beta_{T}$) and uncorrected proper time (τ) were extracted from Pb–Pb collisions at 2.76 TeV; *p*–Pb collisions at 5.02 TeV; Xe–Xe collisions at 5.44 TeV; and *p*–*p* collisions at 0.9, 2.76, 5.02, 7 and 13 TeV. The cited experimental data were measured by the ALICE [29,30,31,32] and CMS Collaborations [33,34]. The changing trends of related parameters with event centrality and collision energy were then obtained and analyzed.

The paper is organized as follows. The formalism and method are briefly described in Section 2. Results and discussion are given in Section 3. Finally, we summarize our main observations and conclusions in Section 4.

## 2. Formalism and Method

High-energy collisions are complex processes in which many emission sources are formed. Sources with the same excitation degree may form a local equilibrium state which can be described by the standard distribution. For different equilibrium states which have different excitation degrees, different temperature parameters may be used. At the same time, we neglect the quantum effect and chemical potential due to their small influences on the $p_{T}$ spectra in high-energy collisions.

According to the invariant phase-space source emission distribution of Schnedermann, Sollfrank and Heinz (SSH) [19], Tomášik, Wiedemann and Heinz [35] further developed the blast-wave model with fluctuations. In this model the invariant momentum distribution is calculated by integrating over the space-time coordinates of the source function. According to [35,36], the blast-wave model with fluctuations results in the invariant momentum distribution as follows:

(1) $$\begin{array}{cc} E\frac{d^{3}N}{dp^{3}}= & \frac{\tau m_{T}}{4\pi^{2}\hbar^{3}}\cosh y\int_{0}^{R}rdrG\left(r\right)e^{\mu_{0}/T_{0}}\\ \; & \times I_{0}\left[\frac{p_{T}}{T_{0}}\sinh\eta_{T}\left(r\right)\right]\int_{\eta_{smin}}^{\eta_{smax}}d\eta_{s}\cosh\eta_{s}H\left(\eta_{s}\right)\\ \; & \times \exp\left[-\frac{m_{T}}{T_{0}}\cosh y\cosh\eta_{T}\left(r\right)\cosh\eta_{s}\right], \end{array}$$

where τ is the proper time considered by us as an uncorrected quantity, $m_{T}=\sqrt{p_{T}^{2}+m_{0}^{2}}$ is the transverse mass, $m_{0}$ is the rest mass, $\mu_{0}$ is chemical potential—which is chosen to be 0 at high energy, $T_{0}$ is the kinetic freeze-out temperature of emission source, $I_{0}$ is the modified Bessel functions of the first kind,

(2) $$\begin{array}{c} \eta_{T}\left(r\right)=\frac{1}{2}\ln\frac{1+\beta (r)}{1-\beta (r)} \end{array}$$

is the transverse flow rapidity, $\beta \left(r\right)=\beta_{S}{\left(r/R\right)}^{n_{0}}$ is a self-similar flow profile, $\beta_{S}$ is the flow velocity on the surface of the thermal source, *r* and *R* are, respectively, the radial position and its maximum in the thermal system, $\eta_{s}$ is the source rapidity, $n_{0}$ is a free parameter which is customarily chosen to be 2 [19] due to the quadratic profile resembling the solutions of hydrodynamics closest [37] and G(r) and $H(\eta_{s})$ are, respectively, the transverse and longitudinal source distributions. Generally, transverse flow velocity is $\beta_{T}=\left(2/R^{2}\right)\int_{0}^{R}r\beta \left(r\right)dr=2\beta_{S}/\left(n_{0}+2\right)$. In the case of $n_{0}=2$ as used in [19], we have $\beta_{T}=0.5\beta_{S}$ [38]. $n_{0}$ is not a sensitive quantity. It does not matter if $n_{0}=1$ or $n_{0}=2$. When we choose $n_{0}=1$, the results are similar. To be compatible with [35,36], we used $n_{0}=2$.

For a single source emission, one has $\eta_{s}=0$, G(r)=1 and $H(\eta_{s})=1$. In the natural system of units, where ℏ=1, Equation (1) is simplified as follows:

(3) $$\begin{array}{cc} E\frac{d^{3}N}{dp^{3}}= & \frac{\tau }{4\pi^{2}}m_{T}\cosh y\int_{0}^{R}rdre^{\mu_{0}/T_{0}}\\ \; & \times I_{0}\left[\frac{p_{T}}{T_{0}}\sinh\eta_{T}\left(r\right)\right]\\ \; & \times \exp\left[-\frac{m_{T}}{T_{0}}\cosh y\cosh\eta_{T}\left(r\right)\right]. \end{array}$$

The blast-wave model with fluctuations results in the united density function of *y* and $p_{T}$ as follows:

(4) $$\begin{array}{cc} \frac{d^{2}N}{dydp_{T}}= & \frac{\tau }{2\pi }p_{T}m_{T}\cosh y\int_{0}^{R}rdre^{\left(\mu_{0}/T_{0}\right)}\\ \; & \times I_{0}\left[\frac{p_{T}}{T_{0}}\sinh\eta_{T}\left(r\right)\right]\\ \; & \times \exp\left[-\frac{m_{T}}{T_{0}}\cosh y\cosh\eta_{T}\left(r\right)\right]. \end{array}$$

According to the general expression of invariant momentum, the normalized factor in Equation (1) is $\tau /4\pi^{2}=gV/{\left(2\pi \right)}^{3}$. For pseudoscalar particles $\pi^{+}$, $\pi^{-}$, $K^{+}$ and $K^{-}$, the degeneracy factor is g=1. For *p* and $\bar{p}$, we have g=2 due to their spin being 1/2. For sphere volume, $V=\left(4/3\right)\pi R^{3}$, where *R* is the maximum range of light particles at the kinetic freeze-out. Then, we obtain the real or corrected proper time $\tau_{0}={\left(1.5\tau \right)}^{1/3}$ for emissions of $\pi^{+}$, $\pi^{-}$, $K^{+}$ and $K^{-}$; and $\tau_{0}={\left(0.75\tau \right)}^{1/3}$ for emissions of *p* and $\bar{p}$.

## 3. Results and Discussion

**Figure 1:**
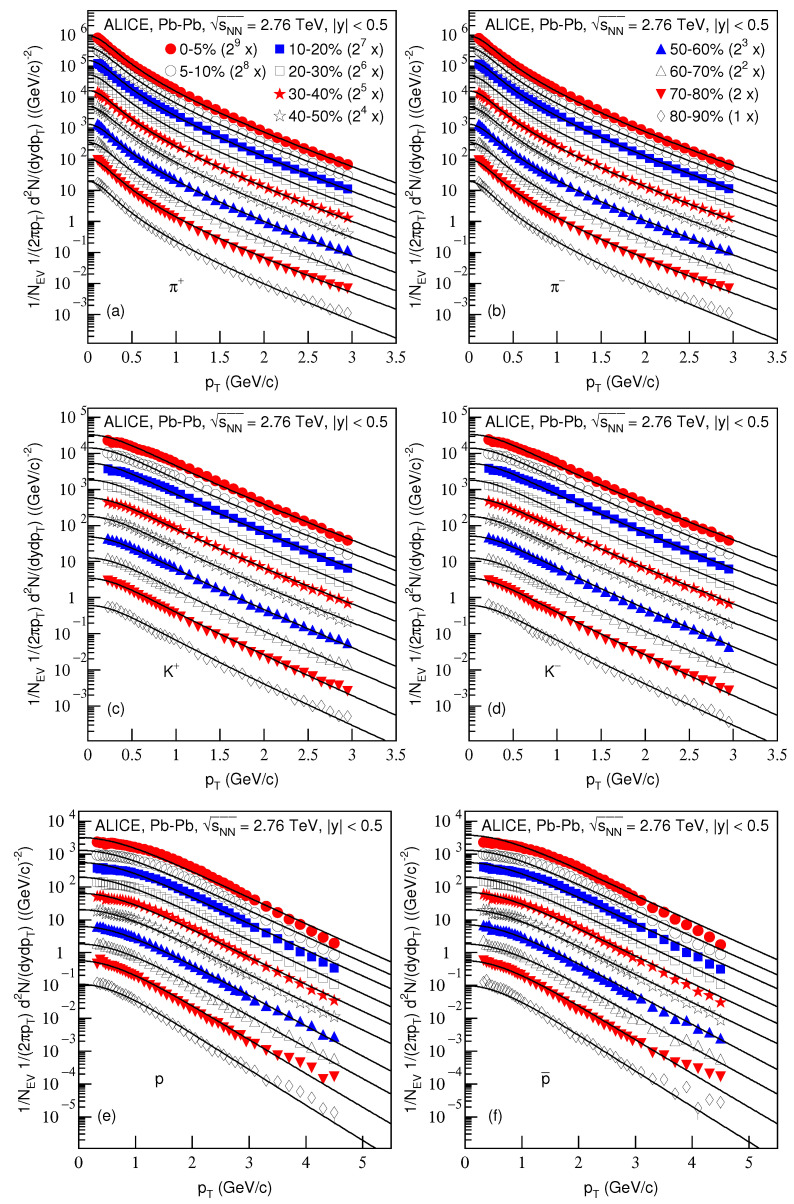
Transverse momentum spectra of (**a**) $\pi^{+}$, (**b**) $\pi^{-}$, (**c**) $K^{+}$, (**d**) $K^{-}$, (**e**) *p* and (**f**) $\bar{p}$ produced in Pb–Pb collisions at $\sqrt{s_{NN}}=2.76$ TeV in the rapidity range |y|<0.5. The closed (open) symbols represent the experimental data measured by the ALICE Collaboration in centralities 0–5%, 5–10%, 10–20%, 20–30%, 30–40%, 40–50%, 50–60%, 60–70%, 70–80% and 80–90% [29]; the spectra for the same particle in different centralities were multiplied by the factor of $2^{n}$ for the clarity—*n* changed from 9 to 0 with the event centrality changing from 0–5% to 80–90%. The curves are our fitting results obtained by using the blast-wave model with fluctuations, Equation (4).

Figure 1 presents the transverse momentum spectra, ($1/N_{EV}){\left(2\pi p_{T}\right)}^{-1}d^{2}N/\left(dydp_{T}\right)$, of (a) $\pi^{+}$, (b) $\pi^{-}$, (c) $K^{+}$, (d) $K^{-}$, (e) *p* and (f) $\bar{p}$ produced in Pb–Pb collisions in different centralities at $\sqrt{s_{NN}}=2.76$ TeV in the rapidity range |y|<0.5. $N_{EV}$ which is usually omitted on the vertical axis denotes the number of events. The symbols represent the experimental data measured by the ALICE Collaboration in different centralities [29] and the curves are our fitting results by using the blast-wave model with fluctuations, i.e., Equation (4). In the fitting, the method of least squares was used to get the minimized $\chi^{2}$. The substantially near event centralities, the values of free parameters’ kinetic freeze-out temperature $T_{0}$, the transverse flow velocity $\beta_{T}$ and proper time τ; and the $\chi^{2}$ degrees of freedom (dof) and corrected proper time $\tau_{0}$ corresponding to the fit are listed in Table 1. In some cases, the $\chi^{2}$ is large, which indicates that the fitting was qualitative and approximately acceptable, and the large dispersion between the curve and data exists. In most cases, one can see the good approximate descriptions of the model results for the experimental data of the ALICE Collaboration in the $p_{T}$ spectra of the identified particles produced in different centralities for Pb–Pb collisions at $\sqrt{s_{NN}}=2.76$ TeV.

**Figure 2:**
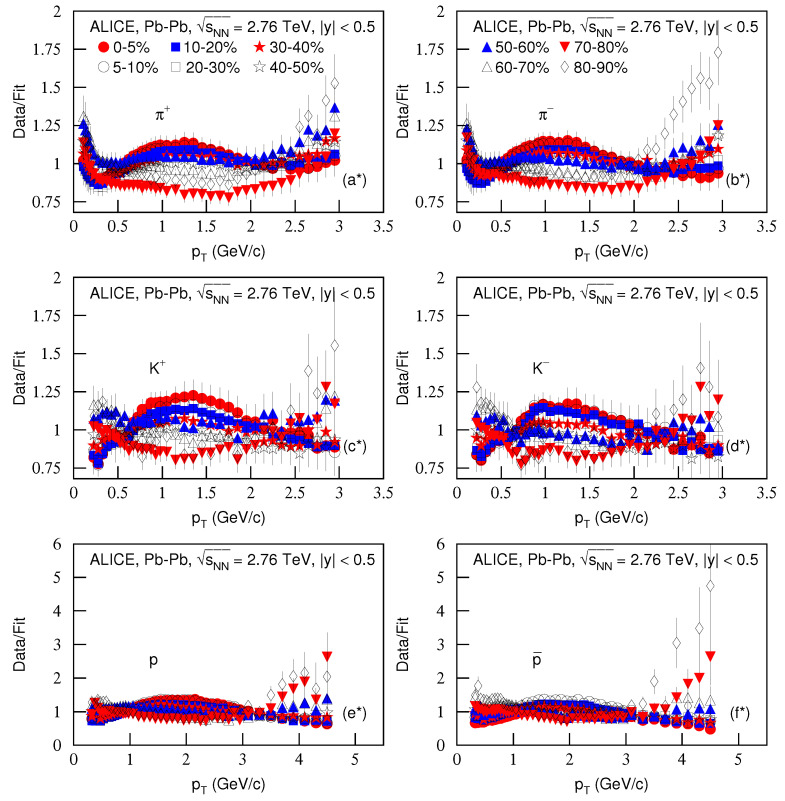
The ratios of data/fit. Panels (a*–f*) correspond to Figure 1a–f, respectively. The different closed (open) symbols represent the data/fit values corresponding to different centralities marked in the Figure 1.

To see the dispersions of the curve from the data, Figure 1 continued (Figure 2) presents the ratios of data/fit. The panels a*–f* correspond to Figure 1a–f, respectively. The different closed (open) symbols represent the data/fit values corresponding to different centralities marked in Figure 2. Indeed, in some $p_{T}$ regions, the ratios are large. This means that the dispersions between the curve and data are large, and the fits are only qualitative and approximate in some cases.

**Figure 3:**
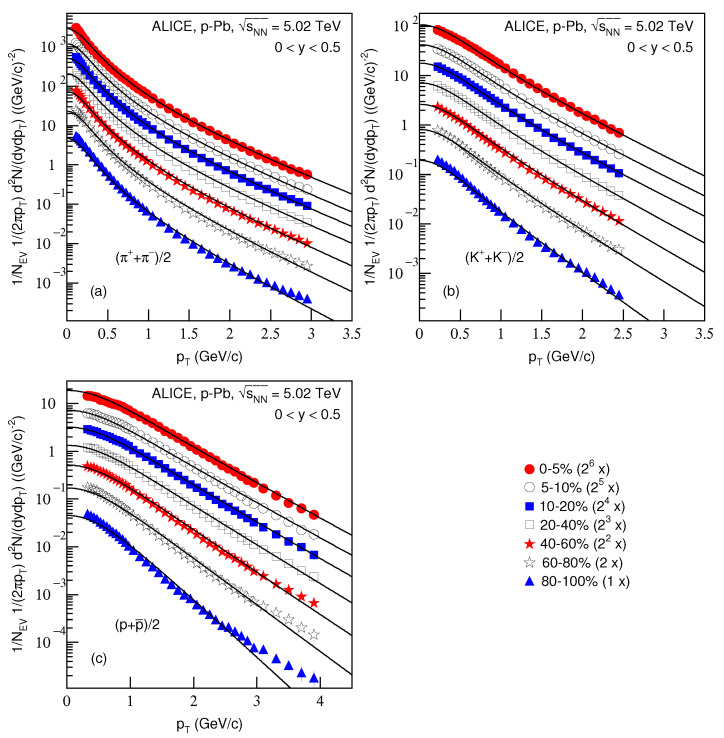
Same as Figure 1, but showing the spectra of (**a**) $(\pi^{+}+\pi^{-})/2$, (**b**) $(K^{+}+K^{-})/2$ and (**c**) $(p+\bar{p})/2$ produced in *p*–Pb collisions at $\sqrt{s_{NN}}=5.02$ TeV in 0<y<0.5. The closed (open) symbols represent the experimental data measured by the ALICE Collaboration in centralities 0–5%, 5–10%, 10–20%, 20–40%, 40–60%, 60–80% and 80–100% [30]. The spectra are scaled by the factor of $2^{n}$, where *n* changes from 6 to 0 with the event centrality changing from 0–5% to 80–100%.

Figure 3 is the same as Figure 1, but it shows the spectra of (a) $(\pi^{+}+\pi^{-}$)/2, (b) $(K^{+}+K^{-})/2$ and (c) $(p+\bar{p})/2$ produced in *p*–Pb collisions at $\sqrt{s_{NN}}=5.02$ TeV in 0<y<0.5. The symbols represent the experimental data measured by the ALICE Collaboration [30]. The related parameters and the centralities are listed together in Table 1. One can see the good approximation the model produced of the experimental data of the ALICE Collaboration in the $p_{T}$ spectra of identified particles produced in different centrality *p*–Pb collisions at $\sqrt{s_{NN}}=5.02$ TeV.

**Figure 4:**
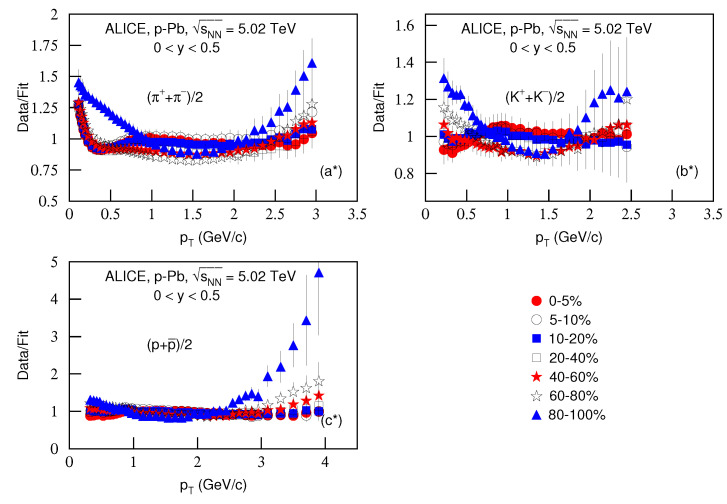
Same as Figure 2, but panels (a*–c*) correspond to Figure 3a–c, respectively.

Figure 4 is the same as Figure 2, but it presents the ratios of data/fit, in which panels a*–c* correspond to Figure 3a–c, respectively. The different closed (open) symbols represent the data/fit values corresponding to different centralities marked in the figure. One can see again that, in some $p_{T}$ regions, the ratios are large, which means large dispersions between the curve and data. In some cases, the fits are only qualitative and approximate.

**Figure 5:**
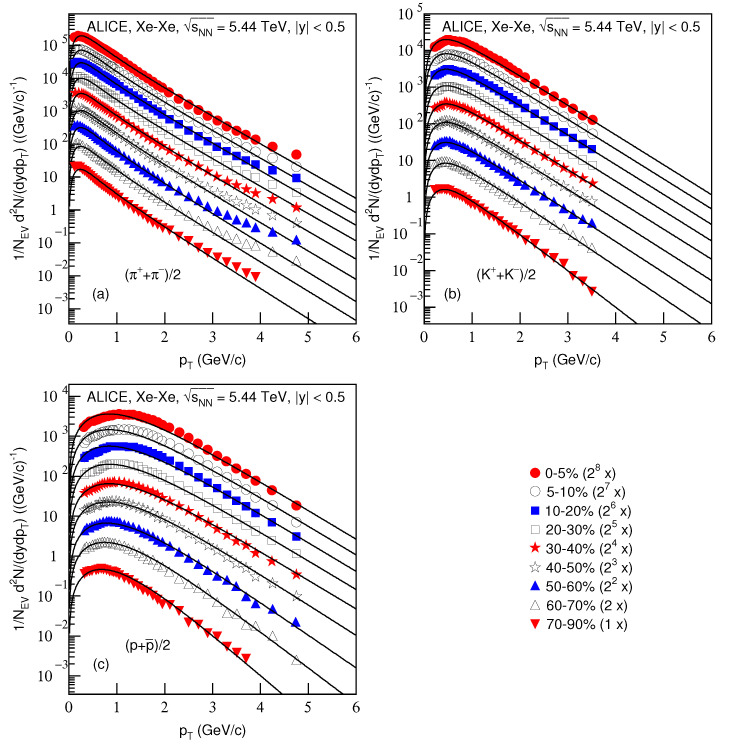
Same as Figure 1, but showing the spectra of (**a**) $(\pi^{+}+\pi^{-})/2$, (**b**) $(K^{+}+K^{-})/2$ and (**c**) $(p+\bar{p})/2$ produced in Xe–Xe collisions at $\sqrt{s_{NN}}=5.44$ TeV in |y|<0.5. The closed (open) symbols represent the experimental data measured by the ALICE Collaboration in centralities 0–5%, 5–10%, 10–20%, 20–30%, 30–40%, 40–50%, 50–60%, 60–70% and 70–90% [31]. The spectra were scaled by the factor of $2^{n}$; *n* changed from 8 to 0 with the event centrality changing from 0–5% to 70–90%.

Figure 5 is the same as Figure 1 and Figure 3, but it shows the spectra, ($1/N_{EV})d^{2}N/\left(dydp_{T}\right)$, of (a) $(\pi^{+}+\pi^{-})/2$, (b) $(K^{+}+K^{-})/2$ and (c) $(p+\bar{p})/2$ produced in Xe–Xe collisions at $\sqrt{s_{NN}}=5.44$ TeV in |y|<0.5. The symbols represent the experimental data measured by the ALICE Collaboration [31]. The related parameters and the centralities are listed together in Table 1. One can see the good approximation the model produced of the experimental data of the ALICE Collaboration in the $p_{T}$ spectra of particles produced in different centrality Xe–Xe collisions at $\sqrt{s_{NN}}=5.44$ TeV.

**Figure 6:**
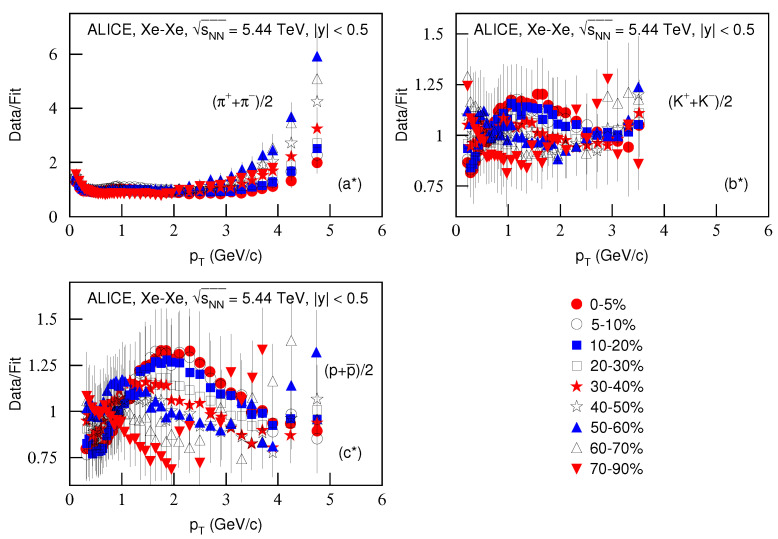
Same as Figure 2, but panels (a*–c*) correspond to Figure 5a–c, respectively.

Figure 6 is the same as Figure 2 and Figure 4, but it presents the ratios of data/fit in which panels a*–c* correspond to Figure 5a–c, respectively. The different closed (open) symbols represent the data/fit values corresponding to different centralities marked in the figure. The same conclusions obtained from Figure 2 and Figure 4 can be obtained from Figure 6.

**Figure 7:**
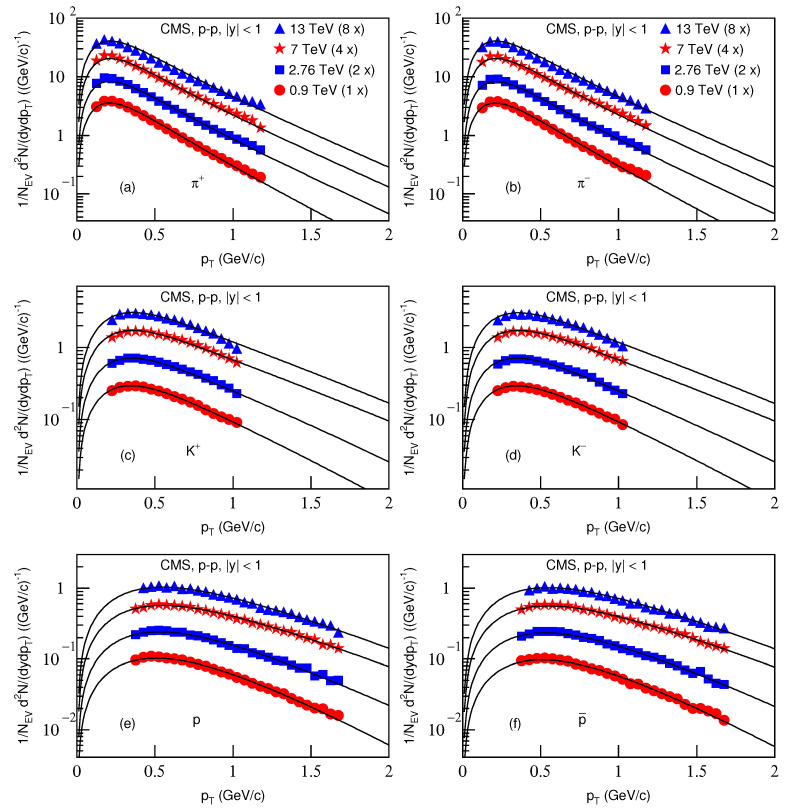
Same as Figure 5, but showing the spectra of (**a**) $\pi^{+}$, (**b**) $\pi^{-}$, (**c**) $K^{+}$, (**d**) $K^{-}$, (**e**) *p* and (**f**) $\bar{p}$, produced in *p*–*p* collisions in |y|<1. The symbols represent the experimental data measured by the CMS Collaboration at $\sqrt{s}=0.9$, 2.76, 7 and 13 TeV [33,34]; the spectra for the same particle in different centralities were multiplied by different amounts shown in the panels for clarity. The spectra were scaled by a factor of $2^{n}$; *n* changed from 0 to 3 with the energy changing from 0.9 to 2.76 TeV.

Figure 7 is the same as Figure 5, but it shows the spectra of (a) $\pi^{+}$, (b) $\pi^{-}$, (c) $K^{+}$, (d) $K^{-}$, (e) *p* and (f) $\bar{p}$ produced in *p*–*p* collisions in |y|<1. The symbols represent the experimental data measured by the CMS Collaboration at $\sqrt{s}=0.9$, 2.76, 7 and 13 TeV [33,34]. The energy and related parameters are listed in Table 2. One can see the good approximation the model produced of the experimental data of the CMS Collaboration in the $p_{T}$ spectra of identified particles produced in *p*–*p* collisions at different energies.

**Figure 8:**
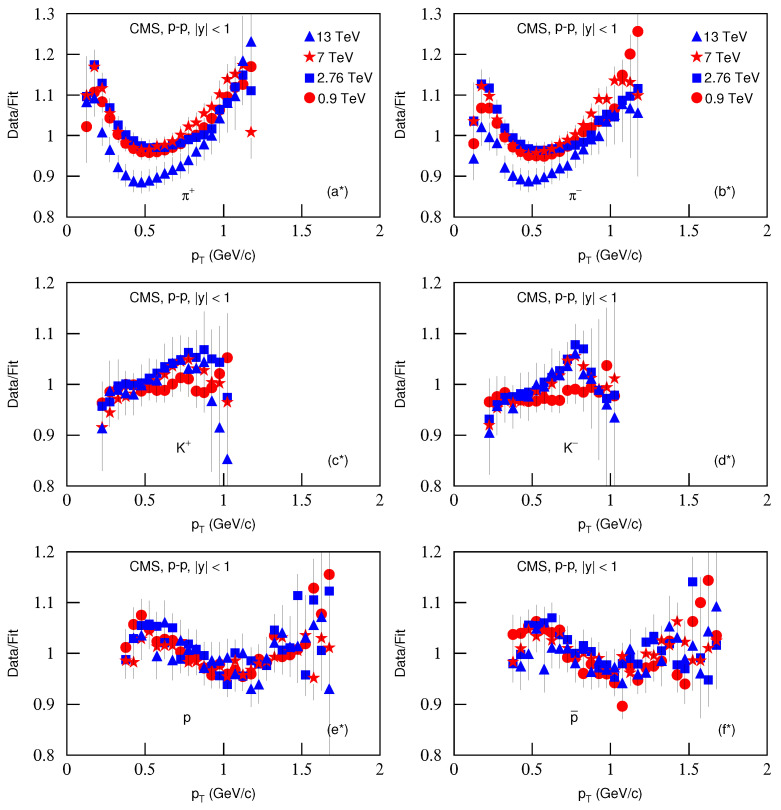
Same as Figure 6, but panels (a*–f*) correspond to Figure 7a–f, respectively.

Figure 8 is the same as Figure 6, but it presents the ratios of data/fit, in which panels a*–f* correspond to Figure 7a–f, respectively. The different closed symbols represent the data/fit values corresponding to different energies marked in the figure. The same conclusions obtained from Figure 2, Figure 4 and Figure 6 can be obtained from Figure 8.

**Figure 9:**
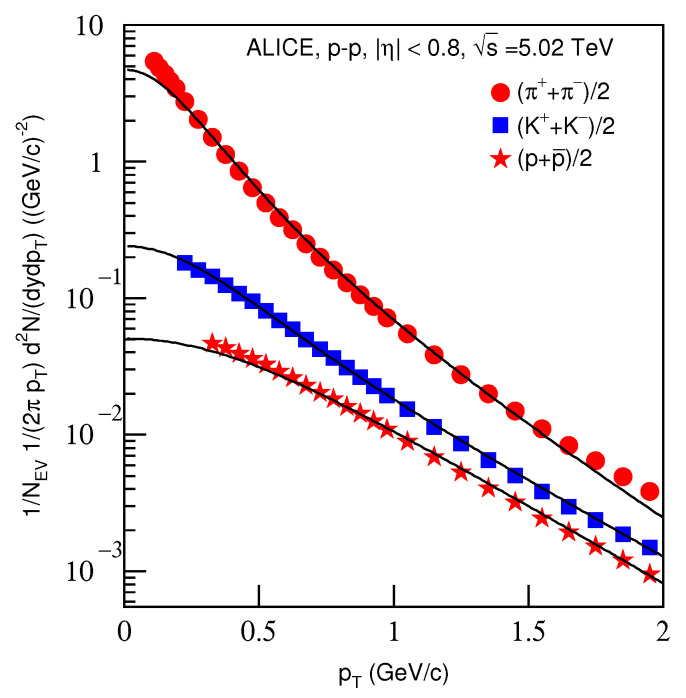
Same as Figure 1, but showing the spectra of $(\pi^{+}+\pi^{-})/2$, $(K^{+}+K^{-})/2$ and $(p+\bar{p})/2$ produced in *p*–*p* collisions in the pseudorapidity range |η|<0.8. The symbols represent the experimental data measured by the ALICE Collaboration at $\sqrt{s}=5.02$ TeV [32].

Figure 9 is the same as Figure 1, but it shows the spectra of (a) $(\pi^{+}+\pi^{-})/2$, (b) $(K^{+}+K^{-})/2$ and (c) $(p+\bar{p})/2$ produced in *p*–*p* collisions in the pseudorapidity range |η|<0.8. The symbols represent the experimental data measured by the ALICE Collaboration at $\sqrt{s}=5.02$ TeV [34]. The energy and related parameters are listed in Table 2. One can see the good approximation the model produced of the experimental data of the ALICE Collaboration in the $p_{T}$ spectra of identified particles produced in *p*–*p* collisions at $\sqrt{s}=5.02$ TeV.

**Figure 10:**
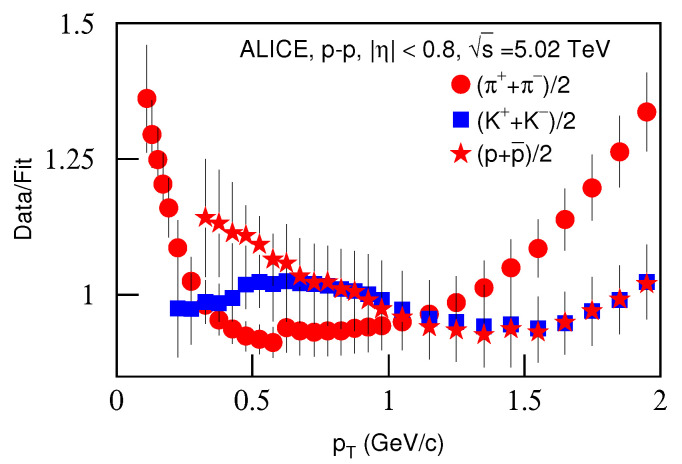
Same as Figure 2, but showing the ratios of data/fit corresponding to Figure 9.

Figure 10 is the same as Figure 2, but it presents the ratios of data/fit corresponding to Figure 9. The different closed symbols represent the data/fit values corresponding to different particles marked in the figure. The same conclusions obtained in the above discussions can be obtained from Figure 10.

From Figure 1, Figure 3, Figure 5, Figure 7 and Figure 9, one can see that we have used the species-dependent parameters to fit the spectra of pions, kaons and protons. This is not the usual way to use the blast-wave model, which fits various spectra simultaneously. We have examined the simultaneous fit of the model for various spectra and know that narrow and different $p_{T}$ ranges have to be used for different particles. We do not think that the simultaneous fitting of various spectra can be better in wide $p_{T}$ ranges. Instead, we may use the individual fit for different spectra and obtain better fits. If needed, we may use the parameters averaged by weighting the yields of different particles to give the simultaneous fit. In fact, the simultaneous fit was not needed in this case, though what we show by the end of this section is a weighted average of proper times for different particles. As will be seen, the individual fit reveals and confirms the mass-dependent differential kinetic freeze-out scenario.

From the above discussions we know that, according to the blast-wave model with fluctuations, we can obtain not only the kinetic freeze-out temperature and transverse flow velocity but also the proper time. The advantage of this model is that the information about proper time can be obtained. This is very important for us to understand the specific evolution of high-energy collisions. The proper time reflects the lifetime of the interacting system. We may discuss the change rule of the proper time by this model. This is an important innovation of the present work. However, the disadvantage of the model is also obvious. In some cases, one component model cannot fit the data well. In fact, it is only applicable to the low-$p_{T}$ region, but not to the high-$p_{T}$ region. This problem is not only a disadvantage in the blast-wave model with fluctuations, but also a disadvantage in all thermal models.

**Figure 11:**
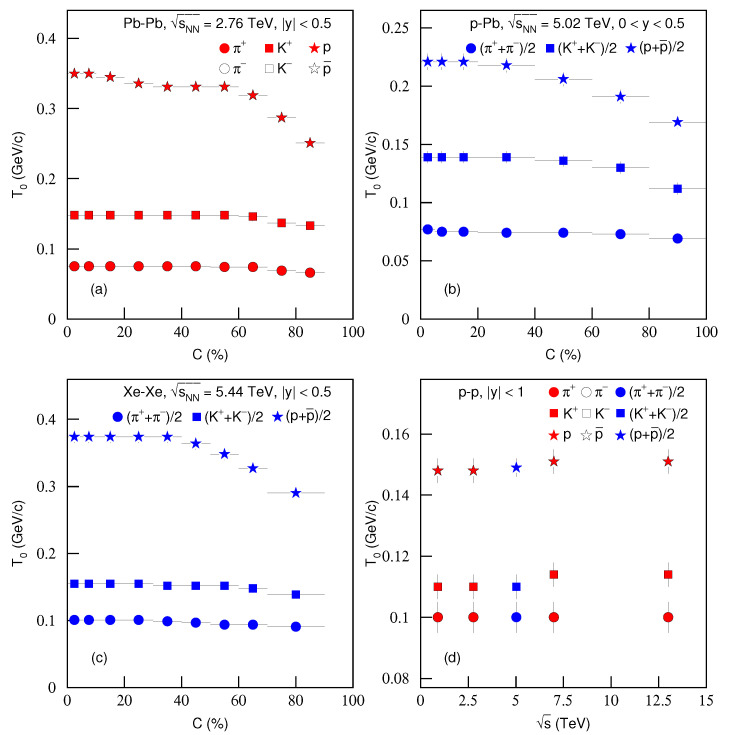
Dependencies of kinetic freeze-out temperature $T_{0}$ on (**a**–**c**) centrality *C* and (**d**) energy $\sqrt{s}$ for the productions of (**a**) $\pi^{+}$, $\pi^{-}$, $K^{+}$, $K^{-}$, *p* and $\bar{p}$ in different centrality Pb–Pb collisions at 2.76 TeV; (**b**) $(\pi^{+}+\pi^{-})/2$, $(K^{+}+K^{-})/2$ and $(p+\bar{p})/2$ in different centrality *p*–Pb collisions at 5.02 TeV; (**c**) $(\pi^{+}+\pi^{-})/2$, $(K^{+}+K^{-})/2$ and $(p+\bar{p})/2$ in different centrality Xe–Xe collisions at 5.44 TeV; and (**d**) $\pi^{+}$, $\pi^{-}$, $K^{+}$, $K^{-}$, *p* and $\bar{p}$ at 0.9, 2.76, 7 and 13 TeV, along with $(\pi^{+}+\pi^{-})/2$, $(K^{+}+K^{-})/2$ and $(p+\bar{p})/2$ at 5.02 TeV. The red closed and black open symbols in panel (**a**) represent positively and negatively charged particles respectively, which are quoted from Table 1. The blue closed symbols in panels (**b**,**c**) represent positively plus negatively charged particles divided by 2, which are quoted from Table 1. The red closed (black open) and blue closed symbols in panel (**d**) represent positively (negatively) and positively plus negatively charged particles divided by 2, which are quoted from Table 2.

To study the change trend of parameters with centrality and energy, Figure 11 shows the dependencies of kinetic freeze-out temperature $T_{0}$ on (a)–(c) centrality *C* and (d) energy $\sqrt{s}$ for the production of different particles in (a) Pb–Pb, (b) *p*–Pb, (c) Xe–Xe and (d) *p*–*p* collisions. Different symbols represent different meanings shown in the panels. One can see that, from peripheral to central collisions, $T_{0}$ slightly increases. The reasons are the more violent interactions in central collisions, where there are higher degrees of excitation, and more participant nucleons being involved in the collisions. $T_{0}$ also increases with the increase of particle mass. This is an evidence of a mass-dependent differential kinetic freeze-out scenario or a multiple kinetic freeze-out scenario [12,39]. In addition, with the increase of energy, $T_{0}$ is nearly invariant at the LHC energies, which implies the nearly saturated density in QGP phase. In *p*–*p* collisions, the dependence of $T_{0}$ on energy is similar to that in peripheral nuclear (A–A and *p*–A) collisions.

**Figure 12:**
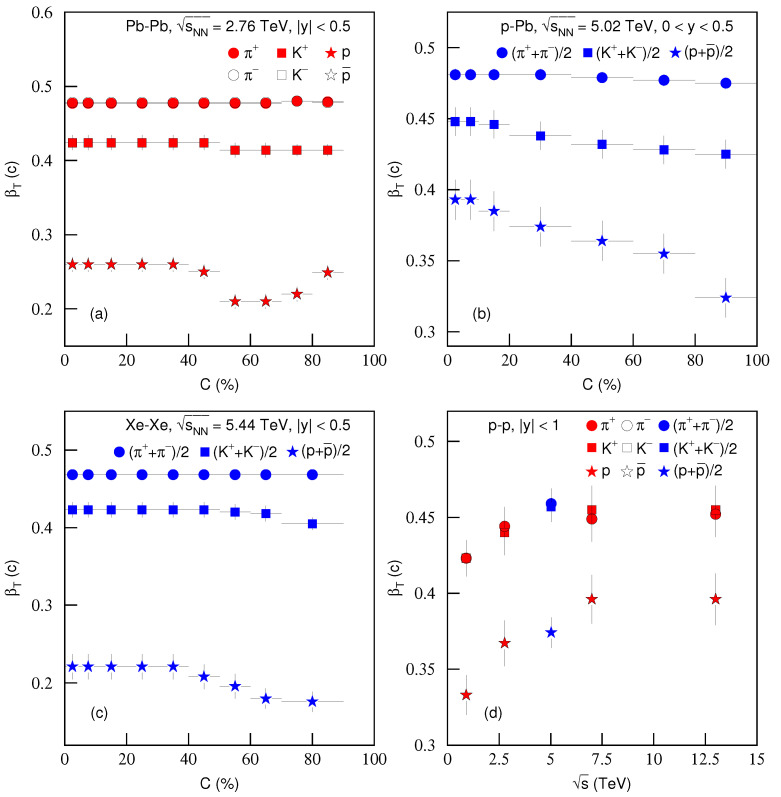
Same as Figure 11, but showing the dependencies of transverse flow velocity $\beta_{T}$ on (**a**–**c**) centrality *C* and (**d**) energy $\sqrt{s}$.

Figure 12 is the same as Figure 11, but it shows the dependence of transverse flow velocity $\beta_{T}$ on (a)–(c) centrality *C* and (d) energy $\sqrt{s}$. Different symbols represent different $\beta_{T}$ of different particles. One can see in general the slight increase of $\beta_{T}$ from peripheral to central collisions. With the increase of energy, $\beta_{T}$ increases slightly. The present work confirms our previous work [27,40,41] which used the intercept-slope method and obtained a slight larger $\beta_{T}$ in central collisions than in peripheral collisions. In our opinion, the flow is produced in the inner core of the interacting system. Even for peripheral or proton–proton collisions, there is non-zero flow velocity. From Figure 12, one can also see the mass-dependent $\beta_{T}$ in most cases. A heavy particle corresponds to a small $\beta_{T}$ due to its large inertia. The differences in $\beta_{T}$ for different particles decrease with the increase of $\sqrt{s}$, where $\beta_{T}$ is large at high $\sqrt{s}$. This reflects that we can neglect the mass effect in a strong flow field.

In the fit process, the free parameters $T_{0}$ and $\beta_{T}$ were not fixed. We looked for the most appropriate parameters according to the values of $\chi^{2}$ in various cases. That is, we obtained the results without bias. As the two parameters were sensitive to the results, they had far less freedom to vary. However, it was inevitable that there would be a correlation between the parameters. The values of $T_{0}$ and $\beta_{T}$ are not independent of centrality classes of up to 60% centrality in nuclear collisions. The same $T_{0}$ and $\beta_{T}$ indicate that the given interaction system has the same excitation degree, expansion characteristic and matter form from 0 to 60% centrality. The same matter can be QGP from the considered central to semi-central collisions.

**Figure 13:**
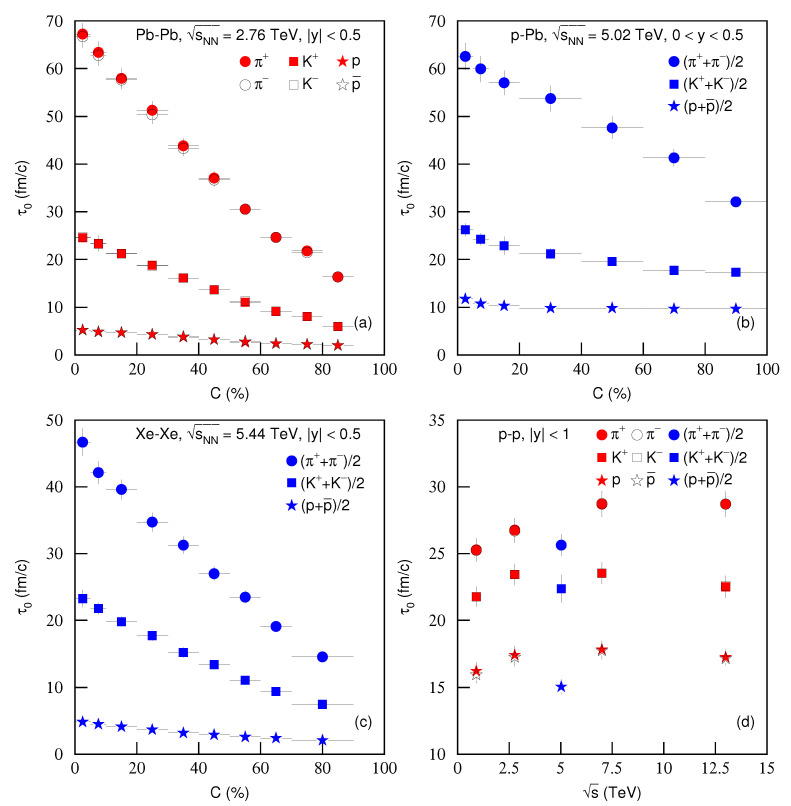
Same as Figure 11, but showing the dependencies of proper time $\tau_{0}$ on (**a**–**c**) centrality *C* and (**d**) energy $\sqrt{s}$.

Figure 13 is the same as Figure 11, but it shows the dependencies of proper time $\tau_{0}$ on (a)–(c) centrality *C* and (d) energy $\sqrt{s}$. Different symbols represent different $\tau_{0}$ for different particles. One can see the increase of $\tau_{0}$ from peripheral to central collisions in panels (a–c), as the number of participant nucleons increases from peripheral to central collisions. Due to the large number of binary collisions by the re-scattering of partons, the system with more participants reached equilibrium slowly. However, the small number of participant nucleons led to the system to go quickly to equilibrium.

Figure 13 also proves the mass-dependent differential scenario from $\tau_{0}$. The heavier the particle is, the smaller $\tau_{0}$ is, which shows the early freeze-out of heavier particles as compared to the lighter particles. This also suggests that different particles have different freeze-out surfaces [39,42]. The result that pions correspond to a much larger $\tau_{0}$ than protons means that the protons stop interacting while pions are still interacting. Due to protons having larger $m_{0}$ than pions, protons are left behind during the system evolution from the origin of collisions in the radial direction, which is the behavior of hydrodynamics [43]. In Figure 13d, one can see, with the increase of energy, that $\tau_{0}$ increases slightly or is nearly invariant. This is consistent with Figure 11d and Figure 12d.

**Figure 14:**
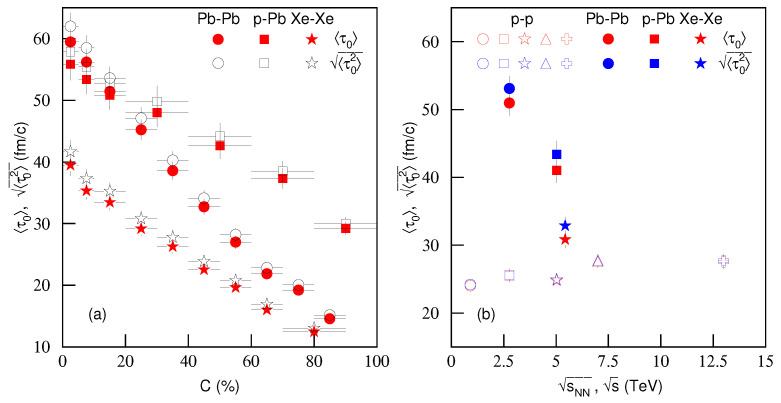
Dependencies of (**a**) average proper time $\langle \tau_{0}\rangle$ and root-mean-square proper time $\sqrt{\langle \tau_{0}^{2}\rangle }$ on centrality *C* and (**b**) $\langle \tau_{0}\rangle$ and $\sqrt{\langle \tau_{0}^{2}\rangle }$ on energy $\sqrt{s_{NN}}$ ($\sqrt{s}$). In panel (**a**), the red closed and black open symbols represent $\langle \tau_{0}\rangle$ and $\sqrt{\langle \tau_{0}^{2}\rangle }$, respectively, from Pb–Pb, *p*–Pb and Xe–Xe collisions, which are listed in Table 3. In panel (**b**), the red and blue open symbols represent $\langle \tau_{0}\rangle$ and $\sqrt{\langle \tau_{0}^{2}\rangle }$, respectively, from *p*–*p* collisions, which are listed in Table 4. The red and blue closed symbols represent $\langle \tau_{0}\rangle$ and $\sqrt{\langle \tau_{0}^{2}\rangle }$ respectively from Pb–Pb, *p*–Pb and Xe–Xe collisions, which are also listed in Table 4.

To study further the dependence of proper time on centrality *C* and energy $\sqrt{s}$, Figure 14a shows the dependencies of the average proper time $\langle \tau_{0}\rangle$ and the average root-mean-square proper time $\sqrt{\langle \tau_{0}^{2}\rangle }$ on *C*. The red closed and black open symbols represent $\langle \tau_{0}\rangle$ and $\sqrt{\langle \tau_{0}^{2}\rangle }$, respectively, which are from Pb–Pb, *p*–Pb and Xe–Xe collisions and are also listed in Table 3 with the impact parameter *b*. These averages were obtained by different particle weights due to different contribution fractions of $\pi^{\pm }$, $K^{\pm }$, *p* and $\bar{p}$. One can see the clear increase of $\langle \tau_{0}\rangle$ and $\sqrt{\langle \tau_{0}^{2}\rangle }$ from peripheral to central collisions. The reasons are the more violent interactions in central collisions in which there is a higher degree of excitation, and the higher number of participant nucleons involved.

Figure 14b shows the dependencies of $\langle \tau_{0}\rangle$ and $\sqrt{\langle \tau_{0}^{2}\rangle }$ on energy $\sqrt{s_{NN}}$ ($\sqrt{s}$). The red and blue open symbols represent $\langle \tau_{0}\rangle$ and $\sqrt{\langle \tau_{0}^{2}\rangle }$, respectively, from *p*–*p* collisions. The red and blue closed symbols represent $\langle \tau_{0}\rangle$ and $\sqrt{\langle \tau_{0}^{2}\rangle }$, respectively, from Pb–Pb, *p*–Pb and Xe–Xe collisions, which were calculated from Table 3 in the range of b=0–8 fm by different weights (∝b) due to different contribution fractions. The cases of large *b* were not included to reduce the influence of the cold nuclear effect. The results for *p*–*p* collisions corresponding to red and blue open symbols were calculated by different particle weights. The values of proper times used in Figure 14b are also listed in Table 4. In *p*–*p* collisions, one can see that with an increase of energy, $\langle \tau_{0}\rangle$ and $\sqrt{\langle \tau_{0}^{2}\rangle }$ are slightly increscent or nearly invariant. In nuclear collisions, at similar energies, the considered proper times for Pb–Pb collisions are larger than those for *p*–Pb collisions, and the latter are larger than Xe–Xe collisions. Generally, nuclear collisions correspond to larger proper times than *p*–*p* collisions.

Figure 14a shows that the order of $\langle \tau_{0}\rangle$ and $\sqrt{\langle \tau_{0}^{2}\rangle }$ is Pb–Pb > *p*–Pb > Xe–Xe in central collisions. However, $\langle \tau_{0}\rangle$ and $\sqrt{\langle \tau_{0}^{2}\rangle }$ in peripheral *p*–Pb collisions are larger than those in peripheral Pb–Pb and Xe–Xe collisions. This is caused by the cold nuclear effect in A–A collisions. In order to avoid the cold nuclear effect, we considered only the weighted average of the events from central to semi-central Pb–Pb and Xe–Xe collisions. This was the case for A–A collisions in Figure 14b, for which one can see the order of $\langle \tau_{0}\rangle$ and $\sqrt{\langle \tau_{0}^{2}\rangle }$ was Pb–Pb > *p*–Pb > Xe–Xe > *p*–*p*. This order is concordant with that of the maximum size between the projectile and target, and that of the other size if the maximum size is the same. This is direct evidence for the statement that the maximum size of the nucleus determines the lifetime of the interacting system, and the other size also affects it. The larger the maximum size, the longer the system lifetime. If the maximum sizes are the same, the larger other size will determine the longer system lifetime.

**Figure 15:**
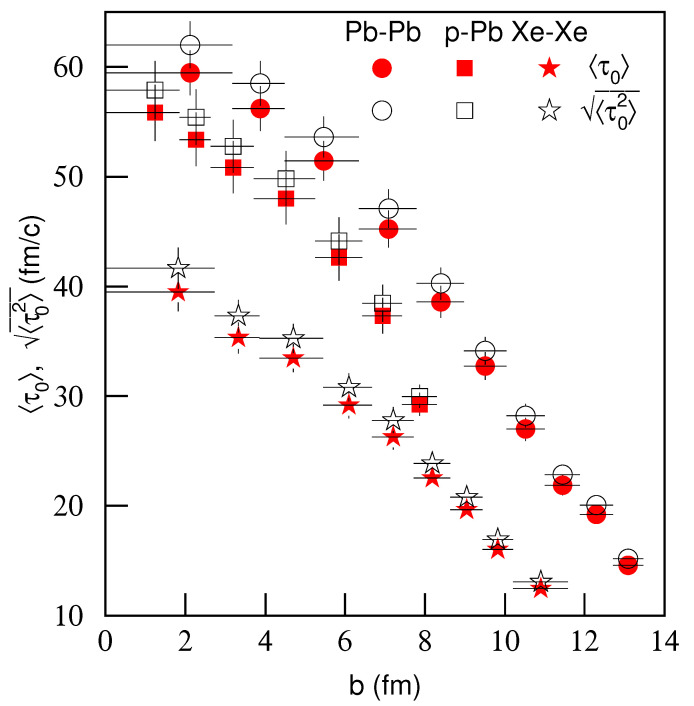
Same as Figure 14a, but showing the dependencies of average proper time $\langle \tau_{0}\rangle$ and root-mean-square proper time $\sqrt{\langle \tau_{0}^{2}\rangle }$ on impact parameter *b*.

To see the common property of the symmetric and asymmetric collisions, Figure 15 shows the dependencies of $\langle \tau_{0}\rangle$ and $\sqrt{\langle \tau_{0}^{2}\rangle }$ on impact parameter *b*. The symbols have the same meaning as Figure 14a. One can see that the large differences in Figure 14a disappear in Figure 15. No matter the symmetric and asymmetric collisions, $\langle \tau_{0}\rangle$ and $\sqrt{\langle \tau_{0}^{2}\rangle }$ decrease with increases of impact parameter *b*. In the plot of $\langle \tau_{0}\rangle$ and $\sqrt{\langle \tau_{0}^{2}\rangle }$ versus impact parameter, there is no obvious difference between symmetric and asymmetric collisions. We may say that, compared with the centrality-dependent proper times, the results of impact parameter-dependent proper times are more concordant among different collisions.

## 4. Summary and Conclusions

We summarize here our main observations and conclusions.

(a) We analyzed the transverse momentum spectra of $\pi^{\pm }$, $K^{\pm }$, *p* and $\bar{p}$ produced in different centrality Pb–Pb collisions at $\sqrt{s_{NN}}=2.76$ TeV, *p*–Pb collisions at $\sqrt{s_{NN}}=5.02$ TeV and Xe–Xe collisions at $\sqrt{s_{NN}}=5.44$ TeV. The experimental data measured by the ALICE Collaborations were approximately fitted by the blast-wave model with fluctuations. Meanwhile, the transverse momentum spectra of $\pi^{\pm }$, $K^{\pm }$, *p* and $\bar{p}$ produced in *p*–*p* collisions at $\sqrt{s_{NN}}=0.9$, 2.76, 5.02, 7 and 13 TeV were analyzed. The experimental data measured by the ALICE and CMS Collaborations were approximately fitted by the model. The kinetic freeze-out temperature, transverse flow velocity and proper time were extracted.

(b) The kinetic freeze-out temperature and transverse flow velocity increase or do not vary with increases of event centrality from peripheral to central collisions, indicating higher excitation degree and greater expansion velocity of the system in central collisions. The average proper time increases with increasing event centrality from peripheral to central collisions, indicating longer lifetime of the system in central collisions. This means that in central collisions, the system needs longer average proper time to reach equilibrium. The heavier particles correspond to shorter proper time, showing the early freeze-out of heavier particles as compared to the lighter particles. From kinetic freeze-out temperature, transverse flow velocity and proper time, the mass-dependent differential kinetic freeze-out scenario or multiple kinetic freeze-out scenario is confirmed.

## Figures and Tables

**Table 1:** Values of parameters ($T_{0}$, $\beta_{T}$ and τ), $\chi^{2}$, dof and $\tau_{0}$ corresponding to the fits of the blast-wave model with fluctuations. The collision types, centrality and particle types are listed for clarity.

Figure	Centrality	Particle	$T_{0}$ (GeV)	$\beta_{T}$ (*c*)	τ (fm/*c*)	$\chi^{2}$/dof	$\tau_{0}$ (fm/*c*)
1a	0–5%	$\pi^{+}$	0.075±0.006	0.477±0.006	203,000.0 ± 20,900.0	50.7/38	67.276±2.309
Pb–Pb	5–10%		0.075±0.004	0.477±0.004	170,000.0 ± 18,000.0	48.8/38	63.413±2.238
	10–20%		0.075±0.004	0.477±0.004	130,000.0 ± 14,000.0	44.3/38	57.989±2.082
	20–30%		0.075±0.004	0.477±0.004	90,000.0 ± 9900.0	50.5/38	51.299±1.881
	30–40%		0.075±0.004	0.477±0.004	56,000.0 ± 6100.0	28.4/38	43.795±1.590
	40–50%		0.075±0.004	0.477±0.004	34,000.0 ± 3700.0	19.5/38	37.084±1.345
	50–60%		0.074±0.004	0.477±0.004	19,000.0 ± 2200.0	44.1/38	30.546±1.179
	60–70%		0.074±0.004	0.477±0.004	10,000.0 ± 1200.0	43.5/38	24.662±0.986
	70–80%		0.069±0.004	0.480±0.004	6900.0±780.0	335.9/38	21.792±0.821
	80–90%		0.066±0.004	0.479±0.004	2900.0±310.0	97.6/38	16.324±0.582
1b	0–5%	$\pi^{-}$	0.075±0.006	0.478±0.006	198,000.0 ± 20,600.0	68.6/38	66.719±2.314
Pb–Pb	5–10%		0.075±0.004	0.478±0.004	165,000.0 ± 17,000.0	58.2/38	62.785±2.156
	10–20%		0.075±0.004	0.478±0.004	128,000.0 ± 13,000.0	43.7/38	57.690±1.953
	20–30%		0.075±0.004	0.478±0.004	85,000.0 ± 9500.0	32.6/38	50.331±1.875
	30–40%		0.075±0.004	0.478±0.004	54,000.0 ± 5900.0	22.2/38	43.267±1.576
	40–50%		0.075±0.004	0.478±0.004	33,000.0 ± 3700.0	15.2/38	36.717±1.372
	50–60%		0.074±0.004	0.478±0.004	19,000.0 ± 2200.0	25.0/38	30.546±1.179
	60–70%		0.074±0.004	0.478±0.004	10,000.0 ± 1200.0	45.0/38	24.662±0.986
	70–80%		0.069±0.004	0.480±0.004	6600.0±700.0	184.1/38	21.472±0.759
	80–90%		0.066±0.004	0.478±0.004	2900.0±310.0	98.3/38	16.324±0.582
1c	0–5%	$K^{+}$	0.148±0.006	0.424±0.010	9900.0±1000.0	87.9/33	24.580±0.828
Pb–Pb	5–10%		0.148±0.006	0.424±0.010	8500.0±1800.0	68.7/33	23.362±1.649
	10–20%		0.148±0.006	0.424±0.010	6400.0±800.0	58.2/33	21.253±0.886
	20–30%		0.148±0.006	0.424±0.010	4400.0±550.0	40.2/33	18.758±0.782
	30–40%		0.148±0.006	0.424±0.010	2800.0±400.0	18.5/33	16.134±0.768
	40–50%		0.148±0.006	0.424±0.010	1700.0±300.0	14.9/33	13.662±0.804
	50–60%		0.148±0.006	0.414±0.010	900.0±110.0	24.9/33	11.052±0.450
	60–70%		0.146±0.006	0.414±0.010	500.0±80.0	20.8/33	9.086±0.485
	70–80%		0.137±0.005	0.414±0.008	350.0±60.0	92.5/33	8.067±0.461
	80–90%		0.133±0.005	0.414±0.008	140.0±40.0	50.3/33	5.944±0.566
1d	0–5%	$K^{-}$	0.148±0.006	0.424±0.010	10,100.0 ± 1100.0	58.3/33	24.744±0.898
Pb–Pb	5–10%		0.148±0.006	0.424±0.010	8400.0±1600.0	55.3/33	23.270±1.477
	10–20%		0.148±0.006	0.424±0.010	6300.0±780.0	46.7/33	21.142±0.873
	20–30%		0.148±0.006	0.424±0.010	4300.0±550.0	25.3/33	18.614±0.794
	30–40%		0.148±0.006	0.424±0.010	2800.0±400.0	13.3/33	16.134±0.768
	40–50%		0.148±0.006	0.424±0.010	1700.0±300.0	14.8/33	13.662±0.804
	50–60%		0.148±0.006	0.414±0.010	960.0±110.0	13.4/33	11.292±0.431
	60–70%		0.146±0.006	0.414±0.010	500.0±80.0	25.8/33	9.086±0.485
	70–80%		0.137±0.005	0.414±0.008	350.0±60.0	95.0/33	8.067±0.461
	80–90%		0.133±0.005	0.414±0.008	140.0±40.0	66.7/33	5.944±0.566
1e	0–5%	*p*	0.350±0.006	0.260±0.010	185.0±22.0	243.0/39	5.177±0.205
Pb–Pb	5–10%		0.350±0.006	0.260±0.010	150.0±28.0	238.1/39	4.827±0.300
	10–20%		0.345±0.006	0.260±0.010	133.0±20.0	160.8/39	4.638±0.232
	20–30%		0.336±0.006	0.260±0.010	102.0±22.0	120.9/39	4.245±0.305
	30–40%		0.331±0.006	0.260±0.010	70.0±17.0	87.9/39	3.744±0.303
	40–50%		0.331±0.006	0.250±0.010	44.0±9.0	68.1/39	3.208±0.219
	50–60%		0.331±0.006	0.210±0.010	26.0±6.0	53.4/39	2.692±0.207
	60–70%		0.319±0.006	0.210±0.010	17.0±3.0	101.5/39	2.336±0.137
	70–80%		0.287±0.006	0.220±0.010	15.0±3.0	222.7/39	2.241±0.149
	80–90%		0.251±0.006	0.249±0.010	10.0±2.0	95.6/39	1.957±0.131
1f	0–5%	$\bar{p}$	0.350±0.006	0.260±0.010	185.0±22.0	174.0/39	5.177±0.205
Pb–Pb	5–10%		0.350±0.006	0.260±0.010	150.0±28.0	197.0/39	4.827±0.300
	10–20%		0.345±0.006	0.260±0.010	133.0±20.0	143.6/39	4.638±0.232
	20–30%		0.336±0.006	0.260±0.010	102.0±22.0	106.2/39	4.245±0.305
	30–40%		0.331±0.006	0.260±0.010	75.0±18.0	81.8/39	3.832±0.307
	40–50%		0.331±0.006	0.250±0.010	44.0±9.0	101/39	3.208±0.219
	50–60%		0.331±0.006	0.210±0.010	29.0±6.0	47.0/39	2.791±0.193
	60–70%		0.319±0.006	0.210±0.010	17.0±3.0	92.1/39	2.336±0.137
	70–80%		0.287±0.006	0.220±0.010	15.0±3.0	144.1/39	2.241±0.149
	80–90%		0.251±0.006	0.249±0.010	10.0±2.0	115.1/39	1.957±0.131
3a	0–5%	($\pi^{+}+\pi^{-})/2$	0.077±0.004	0.481±0.004	163,500.0 ± 22,250.0	78.1/38	62.595±2.839
*p*–Pb	5–10%		0.075±0.004	0.481±0.004	143,500.0 ± 19,680.0	62.3/38	59.930±2.740
	10–20%		0.075±0.004	0.481±0.004	123,500.0 ± 16,550.0	82.3/38	57.006±2.546
	20–40%		0.074±0.004	0.481±0.004	103,500.0 ± 15,500.0	170.6/38	53.746±2.683
	40–60%		0.074±0.003	0.479±0.004	72,000.0 ± 10,650.0	183.1/38	47.622±2.348
	60–80%		0.073±0.003	0.477±0.004	47,000.0 ± 6200.0	277.2/38	41.311±1.817
	80–100%		0.069±0.003	0.475±0.004	22,000.0 ± 2305.0	516.5/38	32.075±1.120
3b	0–5%	($K^{+}+K^{-})/2$	0.139±0.005	0.448±0.010	12,000.0 ± 1805.0	15.8/28	26.207±1.314
*p*–Pb	5–10%		0.139±0.005	0.448±0.010	9500.0±1410.0	5.7/28	24.244±1.199
	10–20%		0.139±0.005	0.446±0.010	8000.0±2005.0	2.7/28	22.894±1.913
	20–40%		0.139±0.005	0.438±0.010	6300.0±965.0	7.4/28	21.142±1.079
	40–60%		0.136±0.005	0.432±0.010	5000.0±670.0	35.0/28	19.574±0.874
	60–80%		0.130±0.005	0.428±0.010	3700.0±448.0	50.0/28	17.705±0.715
	80–100%		0.112±0.005	0.425±0.010	3450.0±410.0	125.9/28	17.297±0.685
3c	0–5%	($p+\bar{p})/2$	0.221±0.007	0.393±0.014	2150.0±291.0	41.8/36	11.726±0.529
*p*–Pb	5–10%		0.221±0.007	0.393±0.014	1650.0±232.0	26.7/36	10.736±0.503
	10–20%		0.221±0.007	0.385±0.014	1445.0±223.0	21.3/36	10.272±0.528
	20–40%		0.218±0.006	0.374±0.014	1245.0±185.0	37.6/36	9.774±0.484
	40–60%		0.206±0.006	0.364±0.014	1245.0±185.0	57.7/36	9.774±0.484
	60–80%		0.191±0.006	0.355±0.014	1195.0±166.0	116.1/36	9.641±0.446
	80–100%		0.169±0.005	0.324±0.014	1195.0±166.0	192.5/36	9.641±0.446
5a	0–5%	($\pi^{+}+\pi^{-})/2$	0.101±0.006	0.468±0.007	67,892.5 ± 8920.0	19.5/38	46.697±2.045
Xe–Xe	5–10%		0.101±0.006	0.468±0.007	49,892.5 ± 6044.0	19.2/38	42.141±1.702
	10–20%		0.101±0.006	0.468±0.007	41,392.5 ± 4678.0	23.9/38	39.598±1.492
	20–30%		0.101±0.006	0.468±0.007	27,892.5 ± 3462.0	40.6/38	34.716±1.436
	30–40%		0.099±0.006	0.468±0.007	20,392.5 ± 2569.0	42.4/38	31.274±1.313
	40–50%		0.097±0.006	0.468±0.007	13,142.5 ± 1581.0	53.0/38	27.014±1.083
	50–60%		0.094±0.000	0.468±0.000	8642.5±1056.0	109.1/38	23.491±0.957
	60–70%		0.094±0.006	0.468±0.007	4642.5±559.0	79.8/38	19.096±0.766
	70–90%		0.091±0.006	0.468±0.007	2042.5±349.0	69.3/38	14.524±0.827
5b	0–5%	($K^{+}+K^{-})/2$	0.155±0.006	0.423±0.010	8392.5±1418.0	18.1/30	23.263±1.310
Xe–Xe	5–10%		0.155±0.006	0.423±0.010	6892.5±833.0	13.5/30	21.785±0.878
	10–20%		0.155±0.006	0.423±0.010	5192.5±569.0	10.5/30	19.822±0.724
	20–30%		0.155±0.006	0.423±0.010	3692.5±478.0	5.4/30	17.693±0.763
	30–40%		0.152±0.006	0.423±0.010	2342.5±355.0	3.4/30	15.203±0.768
	40–50%		0.152±0.006	0.423±0.010	1606.0±205.0	5.1/30	13.405±0.570
	50–60%		0.152±0.006	0.420±0.010	906.0±121.0	5.4/30	11.077±0.493
	60–70%		0.148±0.006	0.418±0.010	552.5±66.0	13.8/30	9.393±0.374
	70–90%		0.139±0.005	0.405±0.008	277.5±30.0	20.2/30	7.467±0.269
5c	0–5%	($p+\bar{p})/2$	0.374±0.009	0.221±0.016	147.5±23.0	37.9/32	4.800±0.250
Xe–Xe	5–10%		0.374±0.009	0.221±0.016	119.5±22.0	37.6/32	4.475±0.275
	10–20%		0.374±0.009	0.221±0.016	92.5±16.0	30.6/32	4.109±0.237
	20–30%		0.374±0.009	0.221±0.016	65.0±13.0	19.4/32	3.653±0.244
	30–40%		0.374±0.009	0.221±0.016	42.5±10.0	14.1/32	3.171±0.249
	40–50%		0.364±0.009	0.208±0.016	32.5±6.5	11.0/32	2.899±0.193
	50–60%		0.348±0.009	0.196±0.016	22.5±5.5	13.8/32	2.565±0.209
	60–70%		0.327±0.007	0.180±0.013	18.5±3.5	17.5/32	2.403±0.152
	70–90%		0.290±0.006	0.176±0.013	12.5±2.5	41.7/29	2.109±0.141

**Table 2:** Values of parameters ($T_{0}$, $\beta_{T}$ and τ), $\chi^{2}$, dof and $\tau_{0}$ corresponding to the fits of blast-wave model with fluctuations. The collision type, energy type and particle type are listed for clarity.

Figure	Energy (TeV)	Particle	$T_{0}$ (GeV)	$\beta_{T}$ (*c*)	τ (fm/*c*)	$\chi^{2}$/dof	$\tau_{0}$ (fm/*c*)
7a	0.9	$\pi^{+}$	0.100±0.005	0.423±0.010	10,785.0 ± 1110.0	84.9/19	25.291±0.868
*p*–*p*	2.76		0.100±0.005	0.444±0.013	12,785.0 ± 1300.0	105.3/19	26.767±0.907
	7		0.100±0.005	0.449±0.015	15,785.0 ± 1590.0	120.3/19	28.715±0.964
	13		0.100±0.005	0.452±0.015	15,785.0 ±1585.0	132.7/19	28.715±0.961
7b	0.9	$\pi^{-}$	0.100±0.005	0.423±0.010	10,725.0 ± 1110.0	117.9/19	25.244±0.871
*p*–*p*	2.76		0.100±0.005	0.444±0.013	12,705.0 ± 1300.0	85.7/19	26.711±0.911
	7		0.100±0.005	0.449±0.015	15,815.0 ± 1590.0	116.0/19	28.733±0.963
	13		0.100±0.005	0.452±0.015	15,785.0 ± 1585.0	113.4/19	28.715±0.961
7c	0.9	$K^{+}$	0.110±0.004	0.423±0.012	6885.0±722.0	3.3/14	21.777±0.761
*p*–*p*	2.76		0.110±0.004	0.440±0.015	8585.0±883.0	21.2/14	23.439±0.804
	7		0.114±0.004	0.455±0.016	8685.0±890.0	20.2/14	23.530±0.804
	13		0.114±0.004	0.455±0.016	7605.0±766.0	5.2/14	22.511±0.756
7d	0.9	$K^{-}$	0.110±0.004	0.423±0.012	6885.0±722.0	16.7/14	21.777±0.761
*p*–*p*	2.76		0.110±0.004	0.440±0.015	8585.0±883.0	23.3/14	23.439±0.804
	7		0.114±0.004	0.455±0.016	8685.0±890.0	22.9/14	23.530±0.804
	13		0.114±0.004	0.455±0.016	7685.0±776.0	6.5/14	22.590±0.760
7e	0.9	*p*	0.148±0.003	0.333±0.013	5688.0±665.0	37.9/24	16.218±0.632
*p*–*p*	2.76		0.148±0.003	0.367±0.015	7044.0±810.0	48.9/24	17.416±0.668
	7		0.151±0.003	0.396±0.016	7544.0±839.0	21.2/24	17.819±0.661
	13		0.151±0.003	0.396±0.017	6844.0±688.0	15.3/23	17.250±0.578
7f	0.9	$\bar{p}$	0.148±0.003	0.333±0.013	5408.0±644.0	66.8/24	15.948±0.633
*p*–*p*	2.76		0.148±0.003	0.367±0.015	6824.0±786.0	37.9/24	17.233±0.662
	7		0.151±0.003	0.396±0.016	7444.0±820.0	23.4/24	17.740±0.651
	13		0.151±0.003	0.396±0.017	6744.0±480.0	11.9/23	17.166±0.407
9	5.02	($\pi^{+}+\pi^{-})/2$	0.100±0.003	0.459±0.010	11,242.5 ± 1055.0	187.6/28	25.644±0.802
		($K^{+}+K^{-})/2$	0.110±0.003	0.457±0.010	7457.5±833.0	13.7/23	22.365±0.833
		($p+\bar{p})/2$	0.149±0.002	0.374±0.010	4542.5±499.0	23.5/21	15.047±0.551

**Table 3:** Values of collision type, centrality, impact parameter *b*, average proper time $\langle \tau_{0}\rangle$ and root-mean-square proper time $\sqrt{\langle \tau_{0}^{2}\rangle }$, where $\langle \tau_{0}\rangle$ and $\sqrt{\langle \tau_{0}^{2}\rangle }$ are calculated due to different particle weights.

Figure	Centrality	*b* (fm)	$\langle \tau_{0}\rangle$ (fm/*c*)	$\sqrt{\langle \tau_{0}^{2}\rangle }$ (fm/*c*)
14a	0–5%	2.117	59.491±2.055	62.021±2.140
Pb–Pb	5–10%	3.867	56.202±2.055	58.501±2.075
	10–20%	5.468	51.435±1.814	53.588±1.877
	20–30%	7.083	45.199±1.687	47.088±1.745
	30–40%	8.391	38.598±1.433	40.247±1.473
	40–50%	9.514	32.736±1.246	34.128±1.271
	50–60%	10.521	26.972±1.046	28.179±1.088
	60–70%	11.443	21.870±0.891	22.814±0.918
	70–80%	12.293	19.225±0.725	20.038±0.740
	80–90%	13.087	14.595±0.562	15.176±0.557
14a	0–5%	1.237	55.816±2.546	57.894±2.633
*p*–Pb	5–10%	2.258	53.393±2.452	55.429±2.539
	10–20%	3.196	50.846±2.377	52.776±2.403
	20–40%	4.520	48.017±2.399	49.828±2.489
	40–60%	5.853	42.627±2.091	44.155±2.172
	60–80%	6.935	37.301±1.635	38.492±1.690
	80–100%	7.866	29.258±1.036	29.974±1.054
14a	0–5%	1.817	39.521±1.784	41.663±1.853
Xe–Xe	5–10%	3.320	35.303±1.433	37.289±1.507
	10–20%	4.698	33.435±1.262	35.271±1.327
	20–30%	6.086	29.178±1.220	30.805±1.278
	30–40%	7.209	26.290±1.135	27.793±1.180
	40–50%	8.179	22.541±0.916	23.879±0.961
	50–60%	9.042	19.634±0.816	20.801±0.852
	60–70%	9.829	16.016±0.647	16.938±0.680
	70–90%	10.900	12.463±0.688	13.075±0.731

**Table 4:** Values of collision type, energy, average proper time $\langle \tau_{0}\rangle$ and root-mean-square proper time $\sqrt{\langle \tau_{0}^{2}\rangle }$, where $\langle \tau_{0}\rangle$ and $\sqrt{\langle \tau_{0}^{2}\rangle }$ were calculated due to different impact parameter weights.

Figure	Energy (TeV)	$\langle \tau_{0}\rangle$ (fm/*c*)	$\sqrt{\langle \tau_{0}^{2}\rangle }$ (fm/*c*)
14b	0.9	24.030±0.861	24.173±0.855
*p*–*p*	2.76	25.488±0.894	25.628±0.890
	5.02	24.801±0.794	24.914±0.795
	7	27.621±0.931	27.779±0.935
	13	27.520±0.942	27.690±0.940
Pb–Pb	2.76	50.967±1.843	53.092±1.898
*p*–Pb	5.02	41.031±1.847	43.379±1.977
Xe–Xe	5.44	30.834±1.277	32.848±1.346

## Data Availability

The data used to support the findings of this study are included within the article and are cited at relevant places within the text as references.

## References

[1] Ivanenko D.D., Kurdgelaidze D.F. (1965). Hypothesis concerning quark stars. Astrophysics.

[2] Itoh N. (1970). Hydrostatic equilibrium of hypothetical quark stars. Prog. Theor. Phys..

[3] Lee T.D., Wick G.C. (1974). Vacuum stability and vacuum excitation in a spin-0 field theory. Phys. Rev. D.

[4] Uphoff J., Fochler O., Xu Z., Greiner C. (2012). RHIC and LHC phenomena with a unified parton transport. Acta Phys. Pol. B Proc. Supp..

[5] Zhong Y., Yang C.B., Cai X., Feng S.Q. (2014). A systematic study of magnetic field in Relativistic Heavy-ion Collisions in the RHIC and LHC energy regions. Adv. High Energy Phys..

[6] Chatterjee S., Das S., Kumar L., Mishra D., Mohanty B., Sahoo R., Sharma N. (2015). Freeze-out parameters in heavy-ion collisions at AGS, SPS, RHIC, and LHC energies. Adv. High Energy Phys..

[7] Hwa R.C. (2015). Recognizing critical behavior amidst minijets at the Large Hadron Collider. Adv. High Energy Phys..

[8] Ma G.L., Nie M.W. (2015). Properties of full jet in High-Energy Heavy-Ion Collisions from parton scatterings. Adv. High Energy Phys..

[9] Adamczyk L., Adkins J.K., Agakishiev G., Aggarwal M.M., Ahammed Z., Alekseev I., Alford J., Aparin A., Arkhipkin D., Aschenauer E.C. (2015). Measurements of dielectron production in Au + Au collisions at $\sqrt{s_{NN}}=200$ GeV from the STAR experiment. Phys. Rev. C.

[10] Xu N. (2014). for the STAR Collaboration. An overview of STAR experimental results. Nucl. Phys. A.

[11] Chatterjee S., Mohanty B., Singh R. (2015). Freezeout hypersurface at energies available at the CERN Large Hadron Collider from particle spectra: Flavor and centrality dependence. Phys. Rev. C.

[12] Chatterjee S., Mohanty B. (2014). Production of light nuclei in heavy-ion collisions within a multiple-freezeout scenario. Phys. Rev. C.

[13] Räsänen S.S. (2016). For the ALICE Collaboration. ALICE overview. EPJ Web Conf..

[14] Floris M. (2014). Hadron yields and the phase diagram of strongly interacting matter. Nucl. Phys. A.

[15] Das S., Mishra D., Chatterjee S., Mohanty B. (2017). Freeze-out conditions in proton-proton collisions at the highest energies available at the BNL Relativistic Heavy Ion Collider and the CERN Large Hadron Collider. Phys. Rev. C.

[16] Huovinen P. (2008). Chemical freeze-out temperature in the hydrodynamical description of Au+Au collisions at $\sqrt{s_{NN}}=200$ GeV. Eur. Phys. J. A.

[17] De B. (2014). Non-extensive statistics and understanding particle production and kinetic freeze-out process from *p_T_*-spectra at 2.76 TeV. Eur. Phys. J. A.

[18] Andronic A. (2014). An overview of the experimental study of quark-gluon matter in high-energy nucleus-nucleus collisions. Int. J. Mod. Phys. A.

[19] Schnedermann E., Sollfrank J., Heinz U. (1993). Thermal phenomenology of hadrons from 200*A* GeV S+S collisions. Phys. Rev. C.

[20] Abelev B.I., Aggarwal M.M., Ahammed Z., Alakhverdyants A.V., Anderson B.D., Arkhipkin D., Averichev G.S., Balewski J., Barannikova O., Barnby L.S. (2010). Identified particle production, azimuthal anisotropy, and interferometry measurements in Au+Au collisions at $\sqrt{s_{NN}}=9.2$ GeV. Phys. Rev. C.

[21] Tang Z.B., Xu Y.C., Ruan L.J., Van Buren G., Wang F.Q., Xu Z.B. (2009). Spectra and radial flow in relativistic heavy ion collisions with Tsallis statistics in a blast-wave description. Phys. Rev. C.

[22] Tang Z.B., Yi L., Ruan L.J., Shao M., Chen H.F., Li C., Mohanty B., Sorensen P., Tang A.H., Xu Z.B. (2013). Statistical origin of constituent-quark scaling in the QGP hadronization. Chin. Phys. Lett..

[23] Jiang K., Zhu Y.Y., Liu W.T., Chen H.F., Li C., Ruan L.J., Tang Z.B., Xu Z.B. (2015). Onset of radial flow in *p*+*p* collisions. Chin. Phys. Lett..

[24] Heiselberg H., Levy A.M. (1999). Elliptic flow and Hanbury-Brown-Twiss correlations in noncentral nuclear collisions. Phys. Rev. C.

[25] Takeuchi S., Murase K., Hirano T., Huovinen P., Nara Y. (2015). Effects of hadronic rescattering on multistrange hadrons in high-energy nuclear collisions. Phys. Rev. C.

[26] Wei H.R., Liu F.H., Lacey R.A. (2016). Kinetic freeze-out temperature and flow velocity extracted from transverse momentum spectra of final-state light flavor particles produced in collisions at RHIC and LHC. Eur. Phys. J. A.

[27] Wei H.R., Liu F.H., Lacey R.A. (2016). Disentangling random thermal motion of particles and collective expansion of source from transverse momentum spectra in high energy collisions. J. Phys. G.

[28] Lao H.L., Wei H.R., Liu F.H., Lacey R.A. (2016). An evidence of mass-dependent differential kinetic freeze-out scenario observed in Pb-Pb collisions at 2.76 TeV. Eur. Phys. J. A.

[29] Abelev B., Adam J., Adamová D., Adare A.M., Aggarwal M.M., Rinella G.A., Agnello M., Agocs A.G., Agostinelli A., Ahammed Z. (2013). Centrality dependence of *π*, *K*, and *p* in Pb-Pb collisions at $\sqrt{s_{NN}}=2.76$ TeV. Phys. Rev. C.

[30] Abelev B., Adam J., Adamová D., Adare A.M., Aggarwal M.M., Aglieri Rinella G., Agnello M., Agocs A.G., Agostinelli A., Ahammed Z. (2014). Multiplicity dependence of pion, kaon, proton and lambda production in *p*-Pb collisions at $\sqrt{s_{NN}}=5.02$ TeV. Phys. Lett. B.

[31] Ragoni S. (2018). Production of pions, kaons and protons in Xe-Xe collisions at $\sqrt{s_{NN}}=5.44$ TeV. arXiv.

[32] Adam J., Adamová D., Aggarwal M.M., Aglieri Rinella G., Agnello M., Agrawal N., Ahammed Z., Ahmad S., Ahn S., Ahn S.U. (2016). Multiplicity dependence of charged pion, kaon, and (anti)proton production at large transverse momentum in *p*-Pb collisions at $\sqrt{s_{NN}}=5.02$ TeV. Phys. Lett. B.

[33] Chatrchyan S., Khachatryan V., Sirunyan A.M., Tumasyan A., Adam W., Aguilo E., Bergauer T., Dragicevic M., Erö J., Fabjan C. (2012). Study of the inclusive production of charged pions, kaons, and protons in pp collisions at $\sqrt{s}$ = 0.9, 2.76, and 7 TeV. Eur. Phys. J. C.

[34] Sirunyan A.M., Tumasyan A., Adam W., Asilar E., Bergauer T., Brandstetter J., Brondolin E., Dragicevic M., Erö J., Flechl M. (2017). Measurement of charged pion, kaon, and proton production in proton-proton collisions at $\sqrt{s_{NN}}=13$ TeV. Phys. Rev. D.

[35] Tomášik B., Wiedemann U.A., Heinz U.W. (2003). Reconstructing the freeze-out state in Pb+Pb collisions at 158 *A*GeV/*c*. Acta Phys. Hung. A.

[36] Ray R.L., Jentsch A. (2019). Phenomenological models of two-particle correlation distributions on transverse momentum in relativistic heavy-ion collisions. Phys. Rev. C.

[37] Schnedermann E., Heinz U. (1993). Relativistic hydrodynamics in a global fashion. Phys. Rev. C.

[38] Kumar L. (2014). for the STAR Collaboration. Systematics of kinetic freeze-out properties in high energy collisions from STAR. Nucl. Phys. A.

[39] Thakur D., Tripathy S., Garg P., Sahoo R., Cleymans J. (2016). Indication of a differential freeze-out in proton-proton and heavy-ion collisions at RHIC and LHC energies. Adv. High Energy Phys..

[40] Lao H.L., Liu F.H., Lacey R.A. (2017). Extracting kinetic freeze-out temperature and radial flow velocity from an improved Tsallis distribution. Eur. Phys. J. A.

[41] Lao H.L., Liu F.H., Li B.C., Duan M.Y. (2018). Kinetic freeze-out temperatures in central and peripheral collisions: Which one is larger?. Nucl. Sci. Tech..

[42] Thakur D., Tripathy S., Garg P., Sahoo R., Cleymans J. (2016). Indication of differential kinetic freeze-out at RHIC and LHC energies. Acta Phys. Polon. Supp..

[43] Sahoo R. (2019). Possible formation of QGP-droplets in proton-proton collisions at the CERN Large Hadron Collider. AAPPS Bull..

